# Hypertriglyceridemia-Induced Acute Pancreatitis in Pregnancy: A Case Report in Anesthetic Management

**DOI:** 10.7759/cureus.69852

**Published:** 2024-09-21

**Authors:** Jasmine Mosavi, Erik Romanelli

**Affiliations:** 1 Anesthesiology, Albert Einstein College of Medicine, Bronx, USA

**Keywords:** covid-19, high-risk pregnancy, hypertriglyceridemia-induced acute pancreatitis, obstetric anesthesia, pancreatitis management

## Abstract

Severe hypertriglyceridemia in pregnancy, defined as triglycerides >1000 mg/dL, is a rare but high-acuity condition that can precipitate several complications for the mother and fetus, in particular hypertriglyceridemia-induced acute pancreatitis (HTGAP). The treatments employed in the management of hypertriglyceridemia-induced acute pancreatitis (HTGAP) have the potential to significantly alter the anesthetic course of an impending or emergent delivery. In this report, we present two cases of HTGAP, one complicated by a concomitant COVID-19 infection and each with a unique approach to anesthetic management in the setting of two very different and cofounded clinical presentations.

## Introduction

The incidence of acute pancreatitis in pregnancy (APIP) is as low as about three in every 10,000 patients, yet it is a condition that presents a significant risk to both the expectant mother and the developing fetus. Gallstones are the most common attributable cause, with hypertriglyceridemia being implicated in about 1-9% of cases [1,2]. The protocols for managing pancreatitis in the context of pregnancy lack specificity but include supportive care with intravenous (IV) fluids, pain management and reduction of triglyceride levels with insulin, fibrates, omega-3 fatty acids, and a low-fat diet. Plasmapheresis has also emerged as a potential adjunctive therapy for patients whose conditions do not resolve with maximal dietary and pharmacologic management [3]. We present two cases of hypertriglyceridemia-induced acute pancreatitis (HTGAP), each with a unique approach to anesthetic management in the setting of two very different clinical presentations. Written patient authorization was obtained from both patients for publication of this report.

These two cases were previously jointly presented as an abstract at the Society of Obstetric Anesthesia & Perinatology Annual Meeting in May 2022.

## Case presentation

Case 1

Patient 1 is a 35-year-old female, gravida 6, para 5, with a history of three prior cesarean deliveries who presented to the emergency department at 26 weeks of gestation with complaints of new-onset intermittent cramping and pelvic pain. The patient had preterm premature rupture of membranes and chronic abruption and was subsequently admitted to the obstetric antepartum service for expectant management.

Following a meal on hospital day five, the patient experienced intractable vomiting episodes and newly endorsed constant, “burning and sharp” epigastric pain with radiation to the back and left shoulder. A fetal ultrasound was performed and noted fetal tachycardia. Initial lab specimens were reported to be lipemic, showing elevated amylase (201 U/L) and lipase (277 U/L), ruling in acute pancreatitis. Liver function tests were normal, and an abdominal ultrasound showed a fatty liver and a grossly normal-appearing pancreas. Triglyceride count and low-density lipoprotein (LDL) cholesterol were > 5680 mg/dl and > 600 mg/dL, respectively, both well outside the reportable range. The patient was made nil per os (NPO); continuous fetal monitoring and aggressive IV fluid resuscitation were initiated. Despite euglycemia, the Endocrinology and Critical Care teams strongly advocated for the initiation of an insulin drip with concurrent dextrose 10% infusion.

On hospital day eight, the patient became increasingly toxic-appearing with episodes of maternal and fetal tachycardia and became refractory to escalating doses of IV patient-controlled analgesics, prompting transfer to surgical intensive care. A right internal jugular (RIJ) catheter was placed, and plasmapheresis was initiated with 2.5 L of 5% albumin and five units of donor plasma replaced. There was a rapid downtrend in triglyceride levels, followed by an improvement in subjective pain scores.

Once maternal triglycerides improved to <1000 mg/dL, the insulin/dextrose drip was terminated, and fenofibrate, omega-3 fatty acids, and a strict low-fat diet were initiated. Triglycerides remained 600-800 mg/dL throughout the remaining duration of her hospitalization.

**Figure 1:**
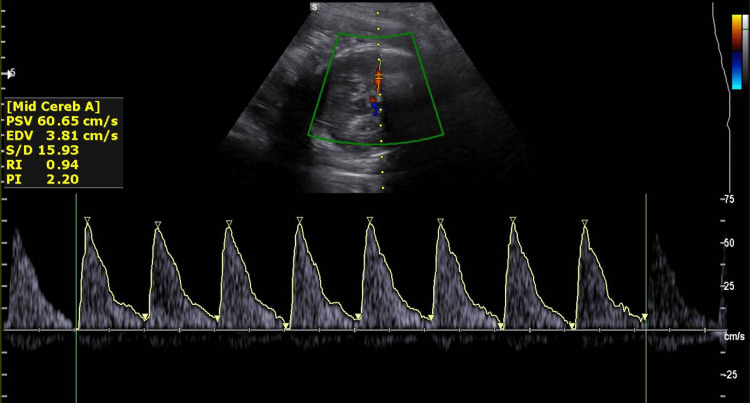
Doppler velocimetry with elevated middle cerebral artery peak systolic velocity indicative of fetal anemia. PSV: peak systolic velocity, EDV: end-diastolic volume, S/D: systolic/diastolic ratio, RI: resistive index, PI: pulsatility index.

On hospital day 17 at 29 weeks gestation, continued fetal tachycardia and follow-up Doppler velocimetry (Figure 1) with elevated middle cerebral artery peak systolic velocity (PSV) raised concern for fetal anemia, prompting expedited delivery. An uncomplicated urgent quaternary cesarean delivery was performed under combined spinal-epidural anesthesia.

**Figure 2:**
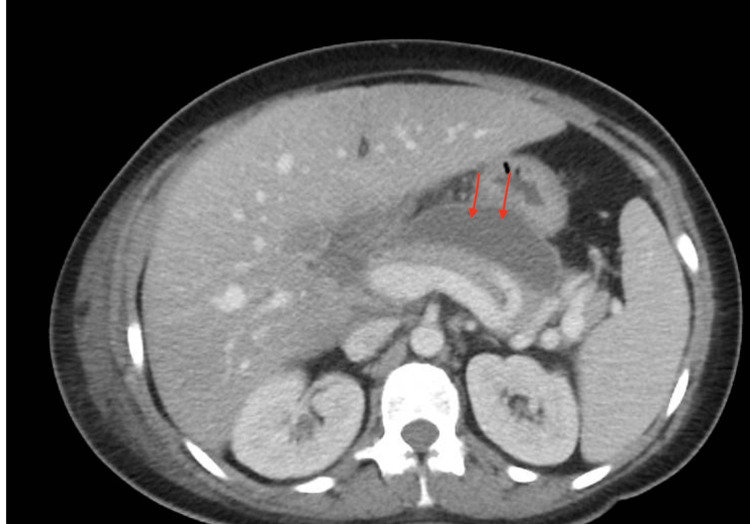
CT abdomen and pelvis noting findings of a collection within the pancreatic less sac measuring approximately 8.3 x 2.5 cm transaxially, suggestive of a pancreatic pseudocyst. CT: computed tomography.

The patient's postpartum course was significant for the development of a left upper extremity deep vein thrombosis provoked by a midline catheter on postoperative day one, which prompted therapeutic enoxaparin administration. A chest computed tomography (CT) was negative for pulmonary embolism but incidentally showed a pancreatic pseudocyst (Figure 2). Gastroenterology did not recommend drainage while inpatient. On the day of discharge, the patient was afebrile, tolerating a regular diet, ambulating freely, and meeting all postoperative milestones. She was discharged with close follow-up and planned genetic testing for familial hypertriglyceridemia.

Case 2

**Figure 3:**
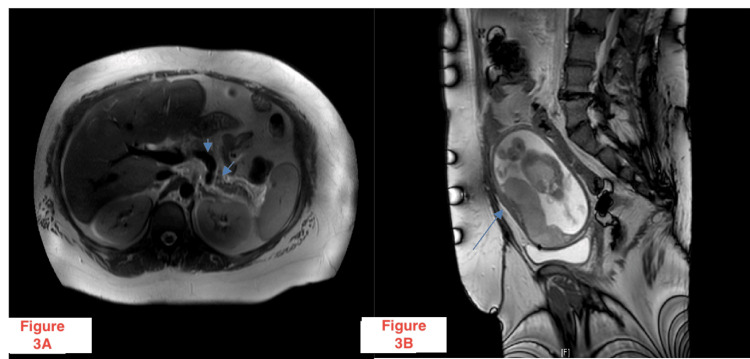
MRI abdomen/pelvis axial (3A) and sagittal (3B) images demonstrating edema of the pancreas with peripancreatic stranding and fluid compatible with pancreatitis.

Patient 2 is a 38-year-old gravida 13, para 1 presenting to the emergency department with complaints of acute abdominal pain, nausea, and multiple episodes of hematochezia. The patient was otherwise unaware but found to be both 20-weeks pregnant and positive for COVID-19. Her history was significant for severe persistent asthma, history of recurrent miscarriages, apolipoprotein CII deficiency, and prior surgical history of gastric bypass, cholecystectomy, and one prior cesarean delivery. Initial labs reported elevated lipase (289U/L). Initially, the patient was stabilized with IV fluids, potassium supplementation, proton pump inhibitors, and patient-controlled analgesia with IV hydromorphone. A central line was placed in the right internal jugular vein due to difficult peripheral vascular access. On further laboratory investigation, triglycerides were found to be 1843 mg/dL, and alanine transaminase (ALT) and aspartate transaminase (AST) were found to be 2622 U/L and 5284 U/L, respectively. Urgent MRI revealed edema of the pancreas with peripancreatic stranding and fluid, resulting in acute pancreatitis (Figures 3A, 3B). Chest X-ray reported left lung atelectasis and infiltrates consistent with early acute respiratory distress syndrome (ARDS). The patient was subsequently admitted to the intensive care unit. 

Despite initial interventions, her condition further deteriorated with the development of sepsis, new-onset coagulopathy (international normalized ratio (INR) rose to 4.4), and acute renal failure, requiring significant transfusion of blood products. Unfortunately, fetal demise was recognized on hospital day 13. The patient subsequently underwent dilation and evacuation (D&E) under general anesthesia. Shortly thereafter, her clinical status improved. The hospital discharged her in stable condition.

## Discussion

Treatment of HTGAP in pregnancy

For the nonpregnant patient, the initial treatment of HTGAP resembles that of any acute pancreatitis patient, with aggressive intravenous (IV) fluid resuscitation, NPO diet, and pain management. Early management with intravenous fluids given at 5-10 mL/kg/h, targeted to a mean arterial pressure of 65-85 mm Hg and urine output >0.5 ml/kg/h, has been shown to result in lower rates of persistent systemic inflammatory response syndrome [1]. Providers should be cognizant of the risks of fluid overload in this setting and employ invasive monitoring and/or point-of-care ultrasound to provide goal-directed therapy. Treatment modalities specific to the management of HTGAP include insulin, heparin, and plasmapheresis. Insulin and heparin therapy lower serum TG levels by increasing the activity of lipoprotein lipase [4,5], and plasmapheresis is a novel but effective method of rapidly removing triglycerides and chylomicrons from the patient’s serum [6].

Anesthetic concerns

When treating HTGAP, a foremost consideration is the effect of the employed treatment modalities on the anesthetic management of impending or emergent delivery. For patient 1, a difficult airway was anticipated due to limited neck range of motion resulting from cervical disc herniations and limited interincisor distance (Mallampati Class IV). Thus, it was emphasized that intubation failure could accompany the usual risks associated with general anesthesia in the pregnant patient (pulmonary embolism, deep vein thrombosis, cardiac arrest, surgical site infection, death) [7], should the patient’s condition rapidly deteriorate and require airway support. In the event of urgent delivery, it was agreed upon that neuraxial anesthesia (NA) should be pursued, barring contraindication. While NA decreases maternal mortality and reduces the risk of thrombotic events, the most serious associated complication is spinal or epidural hematoma, which can portend permanent neurologic damage [8-10]. To reduce this risk, the hemostatic status of the patient must be carefully monitored; thus, in the setting of patient 1, heparin therapy for the treatment of HTGAP was discouraged by anesthesiology unless administered at a low threshold and only if deemed necessary, with abrupt cessation if it became clear that delivery was imminent. Heparin treatment was ultimately forgone in the management of patient 1.

Unlike patient 1, patient 2 was contraindicated for NA due to a new-onset coagulopathy and prothrombin time (PT)/INR of 4.4 that threatened complications of spinal or epidural hematoma. Heparin therapy was also contraindicated for the treatment of pancreatitis due to the significant bleeding risk introduced by coagulopathy. Patient 2 subsequently underwent D&E under general anesthesia following fetal demise.

Patient 2’s accrual of multiple perioperative complications (ARDS, acute kidney failure, sepsis, coagulopathy) introduced special considerations for the D&E performed under general anesthesia. In the setting of acute kidney failure and coagulopathy, it is essential to avoid intraoperative hypotension to maintain renal perfusion. Target mean arterial pressure should be closely monitored, and under- or over-administration of IV fluids should be avoided during fluid resuscitation [11]. Hemodynamic instability in such critically ill patients should be expected; thus, invasive monitoring, vasoactive drugs, and fluids must be readily available [12]. Additionally, ARDS patients should be treated with protective ventilation in order to minimize the risk of ventilator-induced lung injury and improve outcomes [13].

The impact of COVID-19

COVID-19 not only increases the risk for complications in pregnant HTGAP patients by introducing a new avenue for pancreatic injury via the ACE-2 receptor [14], but it also introduces new considerations for the anesthetic management of these patients. Even prior to the COVID-19 pandemic, there have been mounting criticisms regarding the use of general anesthesia in cesarean sections due to the high rate of complications [7]. In the case of patient 2, NA is preferred not only to reduce potential complications, but also to reduce respiratory particle transmission and viral spread during endotracheal intubation and extubation, as well as the pulmonary complications associated with general anesthesia [15]. General anesthesia for cesarean delivery in a COVID-19 patient is indicated only in the setting of maternal respiratory failure, postpartum hemorrhage, or other potentially fatal maternal-fetal indications [15].

## Conclusions

The nuances of anesthetic management in the treatment of APIP underscore the importance of a communicative and collaborative interdisciplinary team in the management of complex cases. The anesthesia team on these cases maintained active involvement in both patients’ ICU care, reviewing the patient’s treatment regimen at each sign-out, and regular communication with the obstetrics and ICU teams was maintained to ensure that the treatment course would support a safe emergent delivery. A multidisciplinary approach is key in such cases during which the treatment of a medical condition can jeopardize the anesthetic management and mortality of mother and fetus.
